# Functional gene networks reveal distinct mechanisms segregating in migraine families

**DOI:** 10.1093/brain/awaa242

**Published:** 2020-09-24

**Authors:** Andreas H Rasmussen, Lisette J A Kogelman, David M Kristensen, Mona Ameri Chalmer, Jes Olesen, Thomas Folkmann Hansen

**Affiliations:** 1 Danish Headache Center, Department of Neurology, Rigshospitalet Glostrup, 2600 Glostrup, Denmark; 2 Novo Nordic Foundation Centre for protein research, Copenhagen University, 2200 Copenhagen, Denmark

**Keywords:** complex trait, gene-gene interaction, genetic network, transcriptomics, migraine

## Abstract

Migraine is the most common neurological disorder worldwide and it has been shown to have complex polygenic origins with a heritability of estimated 40–70%. Both common and rare genetic variants are believed to underlie the pathophysiology of the prevalent types of migraine, migraine with typical aura and migraine without aura. However, only common variants have been identified so far. Here we identify for the first time a gene module with rare mutations through a systems genetics approach integrating RNA sequencing data from brain and vascular tissues likely to be involved in migraine pathology in combination with whole genome sequencing of 117 migraine families. We found a gene module in the visual cortex, based on single nuclei RNA sequencing data, that had increased rare mutations in the migraine families and replicated this in a second independent cohort of 1930 patients. This module was mainly expressed by interneurons, pyramidal CA1, and pyramidal SS cells, and pathway analysis showed association with hormonal signalling (thyrotropin-releasing hormone receptor and oxytocin receptor signalling pathways), Alzheimer’s disease pathway, serotonin receptor pathway and general heterotrimeric G-protein signalling pathways. Our results demonstrate that rare functional gene variants are strongly implicated in the pathophysiology of migraine. Furthermore, we anticipate that the results can be used to explain the critical mechanisms behind migraine and potentially improving the treatment regime for migraine patients.

## Teaser

Using a systems genetics approach, Rasmussen *et al.* identify rare migraine-associated mutations aggregated in modules. The mutations suggest a role in migraine pathophysiology for signalling pathways involving hormones, G-proteins and serotonin receptors.

## Introduction

Migraine is a common neurovascular disorder that is characterized by a unilateral throbbing head pain, accompanied by sensitivity to light and sound and/or nausea/vomiting. It is often accompanied by neurological disturbances (the aura) (Headache Classification Committee of the International Headache Society (IHS), 2018; Szabó *et al.*, 2018). The prevalent types of migraine, migraine with typical aura (MTA) and migraine without aura are considered complex polygenic disorders with a heritability between 40% and 70% (Sutherland and Griffiths, 2017). Scores of common genetic variants have been associated with migraine explaining a part of the genetic variance (Gormley *et al.*, 2016). It has also been shown that the polygenic risk score is higher in families with MTA and migraine without aura; however, it is believed that rare causal variants could also contribute to the genetic variance of migraine (Hansen *et al.*, 2017; Gormley *et al.*, 2018). Such rare causal variants can be detected in families with mendelian-like segregating pattern of migraine, using e.g. whole genome sequencing (WGS). It is encouraging that family studies have identified multiple rare causal variants for a rare dominant form of migraine, familial hemiplegic migraine (Ophoff *et al.*, 1996; Dichgans *et al.*, 2005; Marini *et al.*, 2012; Barros *et al.*, 2013). However, as the prevalent types of migraine are polygenic and do not follow a complete dominant inheritance pattern, we hypothesize that genes with rare variants of small or medium effect size are aggregating in mechanisms specific for migraine. Such mechanisms can be identified in gene networks identified, for example, by RNA sequencing of migraine-relevant tissues in combination with WGS.

Weighted gene co-expression network analysis (WGCNA) (Zhao *et al.*, 2010) has been useful in identifying such gene networks, which has the potential of pointing towards biologically and clinically relevant disease mechanisms (Eising *et al.*, 2017; Feng and Wang, 2017; Liao *et al.*, 2017; Wang *et al.*, 2017). Gene expression is highly tissue-specific, thus co-expression networks of tissues likely to be involved in migraine could suggest migraine-specific networks. The pathophysiology of migraine is not completely understood but it is widely accepted and shown in several studies that the trigeminal ganglion, the visual cortex and the vasculature are involved in critical mechanisms of migraine (Zhang *et al.*, 2010, 2011; Coppola *et al.*, 2013; Brighina *et al.*, 2015; Gormley *et al.*, 2016; Shelukhina *et al.*, 2017; Gaist *et al.*, 2018). The trigeminal ganglion is a cluster of nerve cells controlling sensory input from the face and, for example, due to release of calcitonin gene-related peptide (CGRP) and expression of serotonin 5-HT^d1^, a treatment target of migraine (Potrebic *et al.*, 2003; Messlinger and Russo, 2018). The visual cortex is involved in processing of visual information, and cortical spreading depression in visual cortex explains the aura (Marzoli and Criscuoli, 2017; Charles, 2018). The cardiovascular system has, in addition to specific brain tissues, been linked to migraine (Pickrell *et al.*, 2016). This was recently stipulated as aorta, as common genetic variants associated with migraine were significantly higher expressed in aorta (Gormley *et al.*, 2016).

In this study, we used a systems genetics approach integrating rare genetic variants segregating in migraine families with gene expression networks. We first identified gene networks using WGCNA applied to RNA sequencing data from human aorta (tissue), trigeminal ganglion (tissue) and visual cortex (single nuclei). Second, we integrated rare mutations from our WGS data with gene networks to pinpoint networks having rare variants segregating with migraine, suggesting distinct involvement in migraine mechanisms. Third, these results were replicated in an independent cohort of migraineurs without familial incidences of migraine. The complementary results revealed—for the first time—the critical role of rare gene variants in migraine heritability.

## Materials and methods

**Figure 1:**
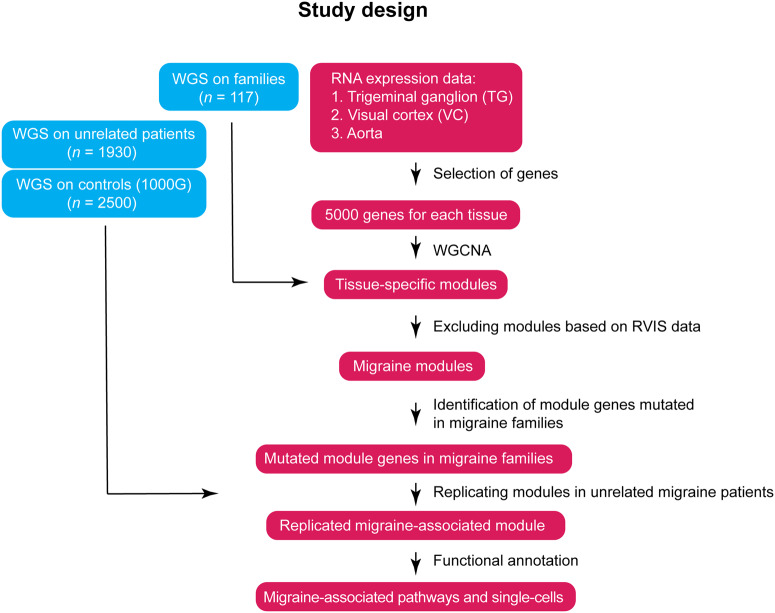
**Study design.** Co-expression networks were constructed for the trigeminal ganglion, visual cortex and aorta, to identify tissue-specific modules of highly co-expressed genes. In parallel, the WGS data were analysed to identify mutations. Modules with over-representations of mutations were defined as potential ‘migraine modules’. Similarly, we defined mutations in an independent cohort compared to population controls and replicated the potential migraine modules. Replicated modules were further analysed using functional annotation. RVIS = residual variation intolerance score.

The study design focused on aorta, trigeminal ganglion and visual cortex as they are key tissues in the pathophysiology of migraine (Zhang *et al.*, 2010, 2011; Coppola *et al.*, 2013; Brighina *et al.*, 2015; Gormley *et al.*, 2016; Shelukhina *et al.*, 2017; Gaist *et al.*, 2018), aiming to identify the pathways involved in the migraine mechanisms (Fig. 1). We constructed co-expression networks of aorta, trigeminal ganglion and visual cortex using RNA sequencing data, for identification of tissue-specific modules using WGCNA. We then integrated data on rare functional variants segregating with migraine, derived from WGS data of 874 individuals from 117 families with migraine, to extracted modules associated with migraine. We aimed to replicate the modules in patients with sporadic migraine (*n *=* *1930) and population control subjects (*n *=* *2500). We then performed functional annotation to map the pathways/networks and cell types involved in migraine.

### Subjects

We recruited 117 families with Mendelian-like segregation of migraine. These families consisted of 874 subjects, 262 of which were diagnosed with MTA, 213 with migraine without aura, 145 with both MTA and migraine without aura, and 254 familial members with no history of migraine. Initially, a migraine patient, i.e. proband, was recruited from the Danish Headache Center and, subsequently, family members were recruited, enabling segregation analysis. The replication cohort consisted of 1930 sporadic migraine patients, 312 of whom were diagnosed with MTA, 1087 were diagnosed with migraine without aura, and 531 were diagnosed with both MTA and migraine without aura; all were recruited from the Danish Headache Center. All participants were assessed by a trained physician or a senior medical student trained in headache diagnostics using a semi-structured interview. The interview was designed by the previous head of classification committee Prof. Jes Olesen to enable diagnosis according to the International Classification of Headache Disorders second edition (ICHD-3) (Olesen, 2006). The study was approved by the Ethics Committee (H-2-2010-122), reported and approved by the data protection agency, and written informed consent was obtained from all participants.

For population controls, we used sequencing data from the 1000 Genomes project (1000G) (Auton *et al.*, 2015). We used the 1000G phase 3 data (VCF files), which included 2500 individuals who were both WGS and whole exome sequenced (WES). When restricting the 1000G cohort to non-Finish Europeans (NFE), 403 individuals were analysed. The NFE classification was provided by 1000G.

### Whole genome sequencing

#### Preparation of samples

Genomic DNA extraction was prepared from whole blood using Illumina TruSeq DNA kit according to the manufacturers’ instructions. In short, 1 µg genomic DNA was isolated from frozen blood and fragmented into 300–400 bp using a Covaris E220 instrument, followed by end repair. AMPure XP magnetic purification beads were then used for size selection followed by PCR enrichment (10 cycles) using appropriate primers. Subsequently, 3′-adenylation and ligation of sequencing adaptors with a T nucleotide overhang was performed and again followed by AMPure magnetic purification. The sequencing libraries were then analysed with regards to quality and concentration using the LabChip® GX (96 samples) instrument from Perkin Elmer. Sequencing libraries were diluted and stored at −20°C. The optimal cluster densities, library insert size, duplication rates and library diversities were assessed by multiplexing on an Illumina MiSeq instrument. All steps in the workflow were monitored using an in-house laboratory information management system (LIMS) with barcode tracking of all samples and reagents.

#### Whole genome sequencing

Sequencing libraries were hybridized to the surface of paired-end flow cells using the Illumina cBot. Paired-end sequencing-by-synthesis was performed on an Illumina HiSeq X instrument. Read lengths used were 2 × 150 cycles of incorporation and imaging. Real-time analysis involved conversion of image data to base-calling in real-time.

#### Reference genome and alignment

The human genome assembly GRCh38 was used as reference sequences map reads. The reference did not include alternate assemblies (GCA_000001405.15_GRCh38_no_alt_analysis_set.fna) in addition to sequences determined to represent common contaminants in the sequencing pipeline. Common contaminants in the sequencing pipeline are the sequences of the bacteriophage PhiX (Enterobacteria_phage_phiX174_sensu_lato_uid14015/NC_001422.fna), the bacteria Ralstonia Pickettii (Ralstonia_pickettii_12D_uid58859) and two sequences from the human microbiome (Coprobacillus_D7_uid32495/NZ_EQ999972.fna and Coprobacillus_D7_uid32495/NZ_EQ999922.fna).

Raw sequences were aligned against the reference with BWA v.0.7.10 (Li and Durbin, 2009). The sequences in the BAM files were realigned around indels with GenomeAnalysisTK- Lite/2.3.9 (McKenna *et al.*, 2010) using a public set of known indels. We marked PCR duplicates with Picard tools v.1.117.

#### Quality control and variant calling

After alignment to reference genome, BAM files were analysed and removed from the analysis based on following criteria: mean base quality >25, per cent marked duplicate >50, mean *n* per read >30, per cent mapping quality below 20 > 11, per cent reads unmapped >40, per cent both reads in a pair unmapped >40, per cent first read in a pair unmapped >40, per cent second read in a pair unmapped >40. Following filtering, variants were called, which resulted in 5 017 453 ± 2607 variants per individual and 697 044 ± 3244 variants with minor allele frequency (MAF) < 5% (Supplementary Fig. 1B and C) and a mean sequencing depth of 32.94× ± 0.22× (Supplementary Fig. 1A).

WGS and variant calling were performed in collaboration with deCODE genetics^®^.

### RNA sequencing data

RNA sequencing data of aorta (*n *=* *247 individuals) were downloaded from GTEx using the R-package recount (Collado-Torres *et al.*, 2017). Sequencing and alignment are described at http://gtexportal.org. In short, RNA sequencing was performed using the Illumina TruSeq library construction protocol, and sequenced on the Illumina Hiseq2000 or HiSeq2500, generating 76-bp paired-end reads. Reads were aligned to the human reference genome (GRCh38) using STAR v.2.5.3a based on the GENCODE v.26 annotation. Read counts and TPM values were produced with RNA-SeQC.

RNA sequencing data of human trigeminal ganglia (*n *=* *16 individuals) was downloaded from NCBI’s Sequence Read Archive (SRX3020382). Messenger RNA was isolated from small subregions of 16 human trigeminal ganglia, as described previously (LaPaglia *et al.*, 2018). In short, non-stranded cDNA libraries were prepared using the Illumina TruSeq RNA Library Preparation Kit v2, and then sequenced on the Illumina HiSeq2500, generating 125-bp paired-end reads. The quality of the fastq-files was checked using the NGSQC toolkit and low quality reads were filtered out according to default settings (cut-off read length of 70% and a cut-off quality score of 20). On average, per sample, 85.2% of the reads were of high quality resulting in on average 26.8 million reads [standard deviation (SD) = 1.9 million reads]. Filtered fastq files were aligned to the human reference genome (Release 32, GRCh38.p13) using the pseudo-aligner Kallisto v.0.46.0. Length-scaled transcripts per million were imported into R using tximport v.3.10.

RNA sequencing data of the human primary visual cortex were downloaded from Allen Human Brain Map (*n *=* *8998) and technical details are described in the technical white paper: transcriptomics (Allen Brain Institute, 2018). In short, 8998 single nuclei were isolated from six cortical layers of visual cortex (*n *=* *8 individuals). The Nextera XT kit (Illumina) was used to prepare cDNA libraries. Single nucleus libraries were then sequenced on the Illumina HiSeq2500, generating 50-bp paired-end reads. Reads were aligned to the human reference genome (GRCh38.p2) using STAR v.2.5.3. Final results included quantification of exon and intron counts. We merged exon and intron data by summing exon and intron counts at gene level.

For all tissues, we included only protein-coded genes, using a gene list extracted from BioMart (GRCh38.p18). This resulted in counts of 19 747, 18 299 and 16 665 genes in trigeminal ganglion, visual cortex and aorta, respectively.

### Construction of co-expression networks

A co-expression network was constructed of each tissue, i.e. aorta, trigeminal ganglion and visual cortex. Genes for network construction were selected based on their connectivity (sum of correlations of a gene with all other genes); the 5000 most highly connected genes per tissue were retained. A network was constructed based on the default pipeline of WGCNA (Zhang *et al.*, 2005; Langfelder and Horvath, 2008). To ensure scale-free topology, the correlation network was raised to a power (beta) to reach a scale-free topology measure (R2) >0.8. To ensure a stable and highly reliable network, the adjacency matrix and consequently dissimilarity topological overlap matrix (TOM) were calculated 100 times by leaving 10% of the samples out every time. The average dissimilarity TOM was calculated from the 100 networks. Next, a gene dendrogram was created and modules containing at least 30 genes were detected using the dynamic tree cutting algorithm. Modules are defined as clusters of highly co-expressed genes. Modules were validated using a permutation procedure as described by Iancu *et al.* (2012). For each network, we randomly selected groups of genes with the same size of the detected modules in the network and computed the average topological overlap. All modules in the aorta, trigeminal ganglion and visual cortex networks had a significantly higher topological overlap than the random modules, except for the ‘grey’ module (which includes unassigned genes). This confirmed that we detected modules with statistical and potentially functional relevance for the tissues.

### Detection of migraine-associated modules

#### Detection of mutated genes in migraine families

We used the pedigree variant annotation, analysis and search tool (pVAAST) v.2.2.0 (Hu *et al.*, 2014) to detect genes with rare functional mutations segregating with migraine. We performed a test for each gene in the modules for each of the 117 families.

Genes were identified with pVAAST based on the following criteria: (i) genes were identified based on the penetrance level (0.9–1) in each family; and (ii) genes were identified based on the functionality of variants only giving rise to frameshift deletions, frameshift insertions, non-frameshift deletions, non-frameshift insertions, non-synonymous single nucleotide polymorphisms (SNPs), splice site mutations, stop-gain and stop-loss mutations. We refer to these functional variants as mutations from here on. Third, genes were identified based on mutations having a MAF < 5% in the genome aggregation database (gnomAD) v.2. See Supplementary Fig. 3A–C for MAF distribution of all variants in the three tissues analysed. The rationale for choosing a MAF < 0.05% was based on the prevalence of migraine being 1:5 to 1:6, biallelic, a genetic heterogeneity of 1, several scenarios of allelic heterogeneity (between 0.01 and 0.05) gave rise to MAF up to 5%, thus a MAF cut-off of 5% was chosen. The cut-off was calculated using the allele frequency app https://www.cardiodb.org/allelefrequencyapp/ (Whiffin *et al.*, 2017).

Genes with rare functional mutations are hereafter termed mutated genes. Genes were termed non-mutated when at least one variant could be detected that was not a rare functional variant.

#### Genic intolerance analysis of modules

The mutation tolerance is gene dependent, thus we calculated the mean residual variation intolerance score (RVIS) (Petrovski *et al.*, 2013) for genes with a rare functional mutation (mutated genes) and for genes without rare functional mutations (non-mutated genes) per module. A one-sided *t*-test was performed to exclude modules where the mutated genes had a significantly higher mean RVIS than the non-mutated genes (*P *<* *0.05).

#### Detection of migraine-associated modules in families

For each tissue, modules with more mutated genes than expected by chance were detected by a one-tailed Fisher’s exact test, i.e. the number of mutated genes versus non-mutated genes in a module against number of mutated genes versus non-mutated genes in all other modules. We only included genes in the Fishers’ exact test in which genetic variations were detectable. Therefore, the number of genes included in Fishers’ exact test for familial migraine were: 4894 in aorta, 4652 in trigeminal ganglion, and 4879 in visual cortex. *P*-values were adjusted using Bonferroni correction for the number of modules (trigeminal ganglion: *n *=* *39, visual cortex: *n *=* *8, aorta: *n *=* *16) and defined as migraine-associated when *P*_adj_ < 0.05.

### Replication of migraine-associated modules in sporadic migraine

Using an independent cohort of 1930 sporadic migraine patients, i.e. with no known history of first-degree relatives with migraine, and using the 1000G control cohort, we assessed the migraine-associated modules found in the migraine family cohort using ANNOVAR (v.2018apr16). ANNOVAR was run using VCF files to annotate genetic variants in mRNAs and to annotate MAF of those genetic variants. We used refSeq and gnomAD v.2 as input databases for ANNOVAR. Module genes with a MAF < 5% were analysed for mutations as defined above. See Supplementary Fig. 3D–F for MAF distribution of mutations. Modules with more mutated genes than expected by chance were determined using one-tailed Fisher’s exact test, i.e. the number of mutated genes versus non-mutated genes in a module against number of mutated genes versus non-mutated genes in all other modules. We only included genes in the Fishers’s exact test in which we could detect genetic variation. Therefore, the number of genes included in the test were for sporadic migraine: 4871 in trigeminal ganglion, 4973 in visual cortex and 4956 in aorta. For 1000G the gene counts were: 3201 in trigeminal ganglion, 2937 in visual cortex and 3251 in aorta. To avoid biases derived from different data processing and batches, the Fisher’s tests were carried out separately for cases and controls. Modules that had significantly more mutated genes in cases but not controls were considered replication.

### Functional annotation of migraine-associated modules

#### Visualization

The Protein-Protein-interaction network was depicted using stringApp v.1.5.0 in Cytoscape v.3.7.2 (Shannon *et al.*, 2003) assessing the protein products and interaction thereof having the threshold for the interaction confidence score > 0.4. Protein interaction data v.11 from www.string-db.org were used as input for Cytoscape.

#### Pathway analysis

We carried out pathway analysis using PANTHER over-representation test (data version released 20190711) (Mi *et al.*, 2013). The total set of genes used for network construction of the tissue of interest was used as reference. Pathways were tested for over-representation using the binomial test and *P*-values were adjusted for multiple-testing using FDR correction and defined as significant in case *P*_adj_ < 0.05.

#### Enrichment analysis in single cell RNA sequencing data

We used two different approaches to investigate modules with increased mutation burden segregating in families. First, we used single cell RNA sequencing data from mouse cortex and hippocampus for cell-type enrichment testing (Zeisel *et al.*, 2015), allowing for differentiation between six different cell types: astrocytes, interneurons, endothelial cells, microglia, oligodendrocytes, pyramidal CA1 neurons and pyramidal SS neurons. Enrichment analysis was carried out using the Expression Weighted Celltype Enrichment (EWCE) R package (Skene and Grant, 2016) with 10 000 bootstraps for genes in the significant migraine-associated modules.

### Data availability

WGS data are considered personal and sensitive data by the scientific ethical committee, thus data can only be shared as part of material agreed upon reasonable request.

## Results

**Figure 2:**
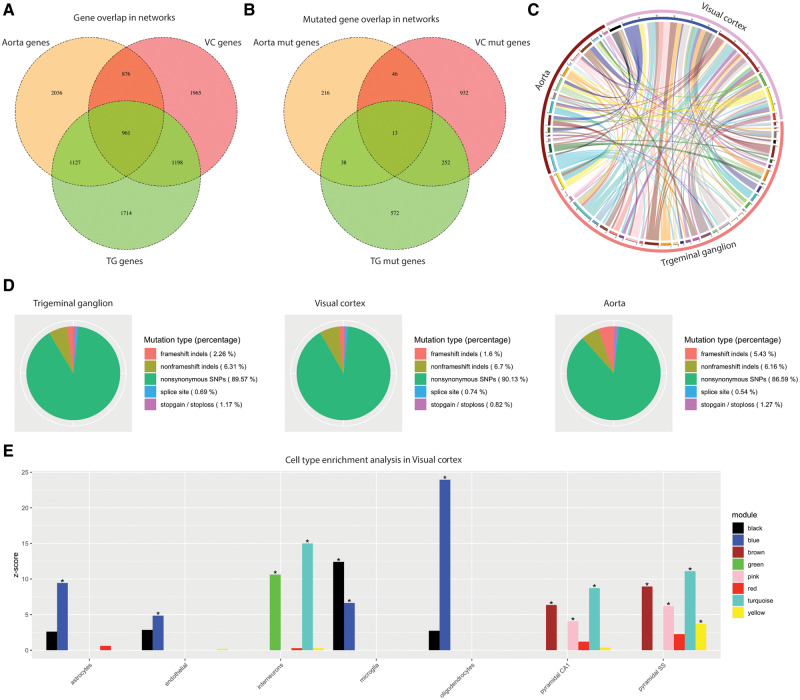
**Overview of genes and mutations in trigeminal ganglion, visual cortex and aorta.** (**A** and **B**) Number of genes and mutated genes in the trigeminal ganglia (TG), visual cortex (VC) network and aorta. (**C**) Module overlap based on the mutated genes in both trigeminal ganglia, visual cortex and aorta. (**D**) Distribution of mutations in mutated genes in trigeminal ganglion, visual cortex and aorta. (**E**) Gene-set enrichment analysis using brain single-cell RNA sequencing data. *P <* *0.05.

We constructed gene co-expression networks for three tissues known to be involved migraine aetiopathology: the aorta, the trigeminal ganglia, and the visual cortex. For each tissue we selected the 5000 most highly connected genes for network construction; 961 genes were present in all three networks (Fig. 2A and Supplementary Table 1). From the derived co-expression networks, we identified 34 modules in the aorta network, 64 modules in trigeminal ganglia and eight modules in visual cortex (Supplementary Fig. 2 and Supplementary Table 1). Though the aorta and trigeminal ganglion networks are based on tissues, the visual cortex network is based on single nuclei data and, therefore, modules may represent subgroups of cell types. Cell type enrichment analysis showed that, for example, the green module was over-represented by genes expressed in interneurons, though other modules (e.g. blue) were covered by different cell types (Fig. 2E).

We used WGS data from our cohort of families with Mendelian-like segregation pattern of migraine, to identify migraine-associated modules, with over-representation of mutated genes. We excluded gene modules that had over-representation of highly mutation-tolerant genes, i.e. type I error (Supplementary Fig. 4). From the aorta network, 16 modules remained for analysis, 39 in trigeminal ganglia and eight in visual cortex.

We identified 313 mutated genes in aorta, 875 mutated genes in trigeminal ganglia, and 1243 mutated genes in visual cortex, of which 13 were present in all three networks (Fig. 2A and B). We found no significant overlap of mutated genes between the three networks (Fig. 2C). The rare mutations consisted primarily of non-synonymous SNPs (86.59–90.13%), while 0.54–5.43% are high impact mutations (frameshift mutations, splice site mutations and stop-loss/stop-gain mutations) (Fig. 2D).

### Selection of migraine-associated modules

**Figure 3:**
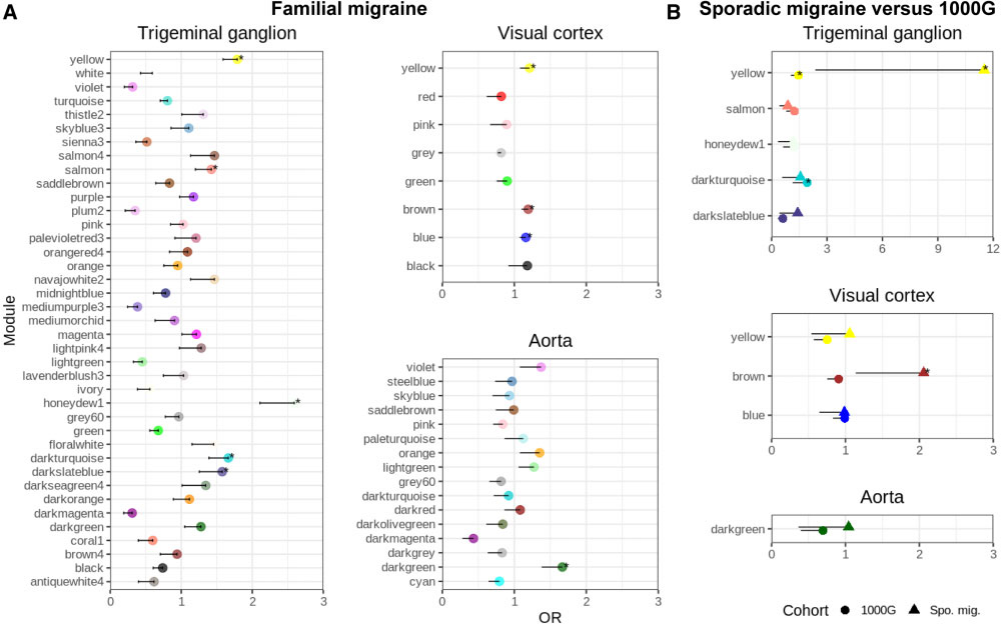
**Identification of migraine-associated modules.** (**A**) Over-representation of mutated genes for familial migraine within modules of the trigeminal ganglia, the visual cortex and in the aorta network. *Modules with an over-representation of mutated genes in familial migraine than are assessed for replication analysis. (**B**) Replication in sporadic migraine (Spo. mig.) compared to 1000G. The odds ratio (OR) is displayed for each module and the error bars show the 95% confidence interval.

As hypothesized, genes with rare mutations are more likely to aggregate in pathways specific for migraine mechanisms. Such genes are likely to be enriched within our constructed networks, based on the expression profile of migraine-relevant tissue. In the aorta network we found one migraine-associated module (Fig. 3A, darkgreen), in the trigeminal ganglion network we found five migraine-associated modules (yellow, salmon, honeydew1, darkturquoise and darkslateblue), and in the visual cortex network we found three migraine-associated modules (blue, brown, yellow) (Fig. 3A).

We were able to replicate the brown module (visual cortex), showing an increased burden of mutated genes in our sporadic migraine cohort and not in the 1000G cohort. We were not able to replicate any of the other modules, although the yellow module (trigeminal ganglia) was significant in the sporadic migraine it was also significantly increased in the 1000G cohort (Fig. 3B). Restricting the 1000G cohort to non-Finish Europeans did not affect the results (Supplementary Fig. 5A–C).

**Figure 4:**
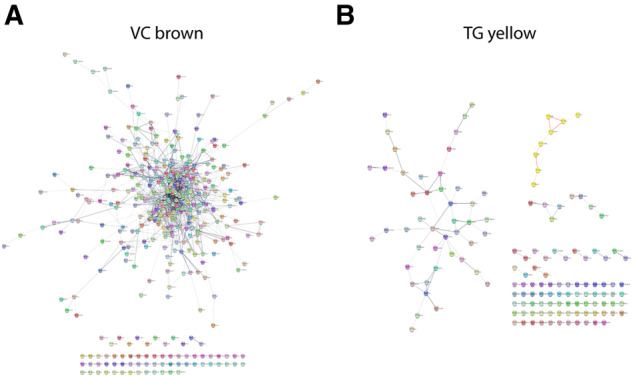
**Analysis of modules on protein level.** (**A**) Network of proteins encoded by genes in the brown module (visual cortex, VC). A large fraction of the proteins contribute to the network. (**B**) Three networks of proteins encoded by genes in the yellow (trigeminal ganglia, TG) module are depicted.

We analysed the visual cortex brown module and the trigeminal ganglion yellow module on a protein level. The majority (82.5%) of the proteins in the visual cortex brown module were participating in the network, suggesting a strong functional module network (Fig. 4A). The trigeminal ganglion yellow module was divided into three smaller networks covering 5.7–32.1% of the proteins, indicating a less strong functional module network (Fig. 4B).

### Functional annotation of migraine-associated modules

**Figure 5:**
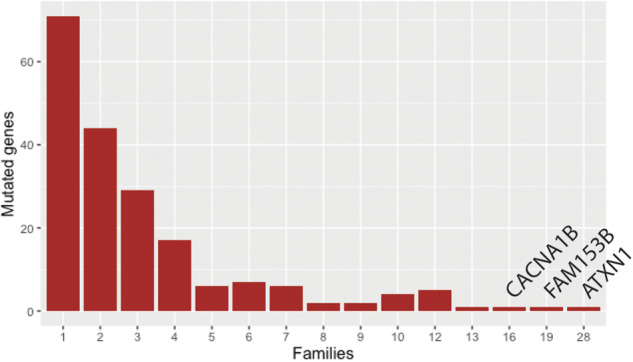
Count of mutated genes in visual cortex brown and in how many families the genes co-segregate with migraine.

Cell type enrichment analysis of the migraine-associated module (visual cortex brown) showed that the module was mainly expressed by interneurons, pyramidal CA1, and pyramidal SS cells (Fig. 2E). Furthermore, five pathways were over-represented: the thyrotropin-releasing hormone receptor (TSHR) signalling pathway, oxytocin receptor (OXTR) mediated signalling pathway, Alzheimer’s disease amyloid secretase pathway, serotonin receptor (5HT_2_R) signalling pathway, and heterotrimeric G-protein signalling pathway-Gq alpha and Go alpha mediated pathway (Table 1). The distribution of mutated genes co-segregating with migraine across families for the module showed that 71 genes have mutations in one family whereas 126 genes harbour functional mutations in two or more families (Fig. 5). Several genes were mutated frequently among families. The top three genes included *ATXN1*, mutated in 28 families; *FAM153B*, mutated in 19 families; and *CACNA1B*, mutated in 16 families.

**Table 1:** Pathways detected in the visual cortex brown module that was over-represented for mutations

Module	Mutated genes	Pathways	*P*-value
VC brown	197	Thyrotropin-releasing hormone receptor signalling pathway	0.046
		Oxytocin receptor mediated signalling pathway	0.045
		Alzheimer’s disease-amyloid secretase pathway	0.040
		5HT2 type receptor mediated signalling pathway	0.040
		Heterotrimeric G-protein signalling pathway-Gq alpha and Go alpha mediated pathway	0.017

Number of mutated genes in families is listed as well as corrected *P*-values.

## Discussion

We present a systems genetics approach integrating WGS data from 117 families with Mendelian-like segregation patterns of migraine with RNA sequencing data from tissues involved in migraine aetiopathology: trigeminal ganglia, visual cortex, and aorta. After replication in an independent cohort of 1930 sporadic cases of migraine, we identified one module with a significant accumulation of mutated genes. The results propose distinct mechanisms affected in migraine, including the TSHR signalling pathway, OXTR signalling pathway, Alzheimer’s disease amyloid secretase pathway, 5HT_2_R signalling pathway and G-protein signalling pathway combined with activity of interneurons, pyramidal CA1 and SS neurons. Further, we were able to suggest specific genes with mutations to be involved in migraine aetiology.

The co-expression approach has been applied to migraine previously. Focusing on common risk variants and using different brain tissues (Eising *et al.*, 2016). The authors allocated migraine-associated genes to modules across brain tissues, showing that oligodendrocytes and gene transcription regulation may play a causal role in migraine. We have shown a similar approach with two important differences. First, our networks are constructed using human RNA sequencing data from the tissues probably involved in migraine aetiopathology, trigeminal ganglia, visual cortex, and aorta. Second, we integrated the co-expression networks with mutations found segregating in a large family cohort.

WGCNA is a well-established method for studying biological networks and diseases. We used it to identify gene modules in three migraine-relevant tissues. Like WGCNA, WGS is also a well-known resource in disease research. Over recent years, the cost of having a genome sequenced has dropped substantially such that it is now possible to sequence entire cohorts (Hayden, 2014). WGS has an advantage over, for example, genotyping since rare variants and *de novo* variants are included in the sequencing. We have—for the first time—utilized both methods in an attempt to reveal more of the defective biological mechanisms leading to migraine. Common variants do not explain 100% of the heritability of migraine (Gormley *et al.*, 2016). Integrating RNA sequencing data via WGCNA with WGS, we have closed some of the gap between how much of the phenotype can be explained by common variants and now by rare mutations (MAF < 0.05%).

Serotonin and the serotonin signalling pathway have, for many years, been known for their implications in migraine. Serotonin levels are believed to increase during migraine attacks (Sicuteri *et al.*, 1961) and the family of serotonin receptor agonists—triptans—are the preferred migraine treatment (Dussor, 2014). Serotonin binds to serotonin receptors, which comprise both metabotropic and ionotropic receptors. The two families of metabotropic serotonin receptors: 5HT_1_ and 5HT_2_, have both been associated with migraine (Hill and Reynolds, 2007; Ferrari *et al.*, 2010; Nelson *et al.*, 2010; Thompson *et al.*, 2012; Yücel *et al.*, 2016) as well as the serotonin transporter (Ogilvie *et al.*, 1998; Marziniak *et al.*, 2005; Bayerer *et al.*, 2010). We directly associate 5HT_2_ with migraine in the visual cortex brown module as well as the G-protein-mediated signalling pathway including G_o_, which signals downstream of the 5HT_1_ receptor. Moreover, we found an association of the visual cortex brown module gene-set to neurons in our EWCE analysis. Cortical neuronal activity initiates and propagates cortical spreading depression (Leao, 1944), which has been linked to serotonergic activity in functional studies (Supornsilpchai *et al.*, 2006; Guedes *et al.*, 2017). We suggest that the migraine phenotype may be caused by mutated genes in the serotonin signalling pathway as well as the downstream G-protein coupled signalling pathway.

In addition to serotonergic neurons, glutamatergic neurons and synapses have also been linked to migraine through different association studies where SNPs in genes related to glutamate transport or signalling have been detected (Shin *et al.*, 2011; Gormley *et al.*, 2016). In addition, glutamate levels have been increased in migraineurs (Ferrari *et al.*, 1990; Martínez *et al.*, 1993; Campos *et al.*, 2013). Glutamate plays a central role in cortical spreading depression as an activator and propagator and inversely, inhibition of glutamate receptors impairs initiation and propagation of cortical spreading depression (Lauritzen and Hansen, 1992; Peeters *et al.*, 2007; Zhou *et al.*, 2013). In our analysis, we detected three genes (*ATXN1*, *FAM153B* and *CACNA1B*) that were mutated in >15 families. *CACNA1B* and *ATXN1* are both involved in glutamate signalling. *CACNA1B* encodes a subunit in a voltage-gated calcium channel, which is expressed at the presynapse and involved in release of glutamate from the presynapse (Bunda *et al.*, 2019). *ATXN1* encodes a DNA binding protein, which is able to regulate expression of the metabotropic glutamate receptor 1 (mGluR1) at the postsynaptic membrane (Notartomaso *et al.*, 2013). Thereby the glutamatergic signalling is altered on both sides of the synaptic cleft in our migraine patients and may contribute to the migraine pathophysiology. *CACNA1B* is a member of a family of genes, which includes *CACNA1A*, that has been linked to a monogenic subtype of MTA, namely familial hemiplegic migraine (FHM) (Ophoff *et al.*, 1996). FHM is a more severe phenotype than MTA and patients with FHM can experience ataxia symptoms. Ataxia is a complex disease and multiple genes have been linked to the disorder including *ATXN1*. The knowledge regarding *FAM153B* is limited and no function of the encoded protein has been reported. According to the GTEX data, *FAM153B* is highly expressed in the brain and likely has a vital function in neurons, which may be affected by mutations (Lonsdale *et al.*, 2013).

Oxytocin and thyrotropin are both hormones that regulate the activity of OXTR and TSHR. Both receptors are G-protein coupled receptors expressed throughout the brain; however, TSHR is expressed in lower amounts than OXTR in the brain according to GTEX data (Lonsdale *et al.*, 2013; Warfvinge *et al.*, 2020). Oxytocin has been the subject of migraine research as it is a strong analgesic in neuroinflammation (Yu *et al.*, 2003; Yang *et al.*, 2007; Engle *et al.*, 2012; Kubo *et al.*, 2017). Moreover, oxytocin is increased during pregnancy, which inversely correlates to the decrease in headaches and migraine of pregnant females (Callaghan, 1968; Kuwabara *et al.*, 1987; Goadsby *et al.*, 2008; Hoshiyama *et al.*, 2012). Oxytocin levels also increase during intercourse and orgasms for both males and females. For the latter, it is reported that 47% experience relief from migraine during sex (Carmichael *et al.*, 1987; Evans and Couch, 2001). Clinical testing of oxytocin in migraine patients led to a long term (24 h) effect on pain after dosing. In the course of 8 weeks, oxytocin had an effect on the migraine frequency (Tzabazis *et al.*, 2017). Thyrotropin is less studied with respect to migraine compared to oxytocin. However, high levels of thyrotropin were associated with lower occurrences of headache (Hagen *et al.*, 2001) and low levels of thyrotropin may be associated with a prolonged and refractory clinical course of migraine (Starikova *et al.*, 2019). Taken together, hormone receptor misregulation seems to play a role in migraine. Our data point to a dysregulation in the hormone receptor pathways, which most likely derives from mutations affecting proteins active downstream from oxytocin and thyrotropin receptors.

Finally, we detected the Alzheimer’s disease amyloid secretase pathway in the visual cortex brown module. It has been suggested that migraine may be associated with mild types of cognitive changes (Araújo *et al.*, 2012) but there is no clear evidence showing whether or not migraine is a risk factor for Alzheimer’s disease (Breteler *et al.*, 1991; Wang *et al.*, 2018; Lee *et al.*, 2019; Morton *et al.*, 2019).

### Strengths and limitations

In this study we used a novel approach by combining gene-gene interaction networks based on RNA sequencing data with rare mutations found in families using WGS data. The co-expression networks enable investigation of biological mechanisms in migraine-relevant tissues. The construction of those networks is affected by the origin and quality of the RNA sequencing data, i.e. inter-tissue comparison is not possible becuase of, for example, their origin, bulk versus single cell, and sequencing depth. The modules revealed in the aorta and trigeminal ganglia data are driven by differences in gene expression levels between individuals, while in the visual cortex network, using single nuclei data, modules are driven by differences in cell types. Interpretation of pathways detected in visual cortex, therefore, needs to be done with caution. Similarly, thresholds applied to mutation calling affect results. We have analysed mutations with a MAF < 0.05 in both familial and sporadic migraine. The recent migraine GWAS included variants with a MAF as low as 0.01 (Gormley *et al.*, 2016). Our study may, therefore, overlap with respect to these variants. However, we believe it is a strength to include variants with a MAF up to 0.05 because of the high prevalence of migraine in the population.

The differences in the number of genes having genetic variants in the families was marginal, which is also reflected by the mean coverage of the genome. The difference between the replication cohorts most likely derives from different sample handling and sequencing, hence the separate assessment of the sporadic migraine cohort and 1000G cohort. Thus, a more ideal comparison of having controls from the same population and process together would have been preferred.

Our familial migraine cohort has a large fraction of migraine patients with aura. This is due to the initial recruiting of our patients and may be considered as a limitation. However, our replication cohort of sporadic migraine has a small fraction of MTA patients compared to migraine without aura patients. Despite the fact that there are no differences in clinical presentation between familial migraine and sporadic migraine, our results may be slightly biased towards MTA since we initially analysed our familial cohort (Ravn *et al.*, 2019). If more familial migraine without aura patients were included in future studies resembling our study, it could perhaps result in more gene modules implicated in the migraine biology.

## Conclusion

Migraine-associated mechanisms derived from a visual cortex module exist and are likely to contribute to the migraine pathophysiology. Our data support previous findings that the serotonin, glutamate, hormonal and G-protein signalling pathways are involved in migraine. We suggest that a dysfunction in neurotransmitter and hormone signalling is part of migraine pathophysiology.

## Supplementary Material

awaa242_Supplementary_Data

## Abbreviations

MAF =: minor allele frequency
MTA =: migraine with typical aura
WGCNA =: weighted gene co-expression network analysis
WGS =: whole genome sequencing
