# Supplementary material for: Functional gene networks reveal distinct mechanisms segregating in migraine families
Source: Brain. 2020 Sep 24;143(10):2945–56. doi: 10.1093/brain/awaa242 (PMC7780491; doi:10.1093/brain/awaa242)
Supplement: awaa242_Supplementary_Data [file awaa242_supplementary_data.zip › awaa242-suppl_data/brain-2019-02160-File008.pdf]

Functional gene networks reveal distinct mechanisms segregating in migraine families

Supplementary data

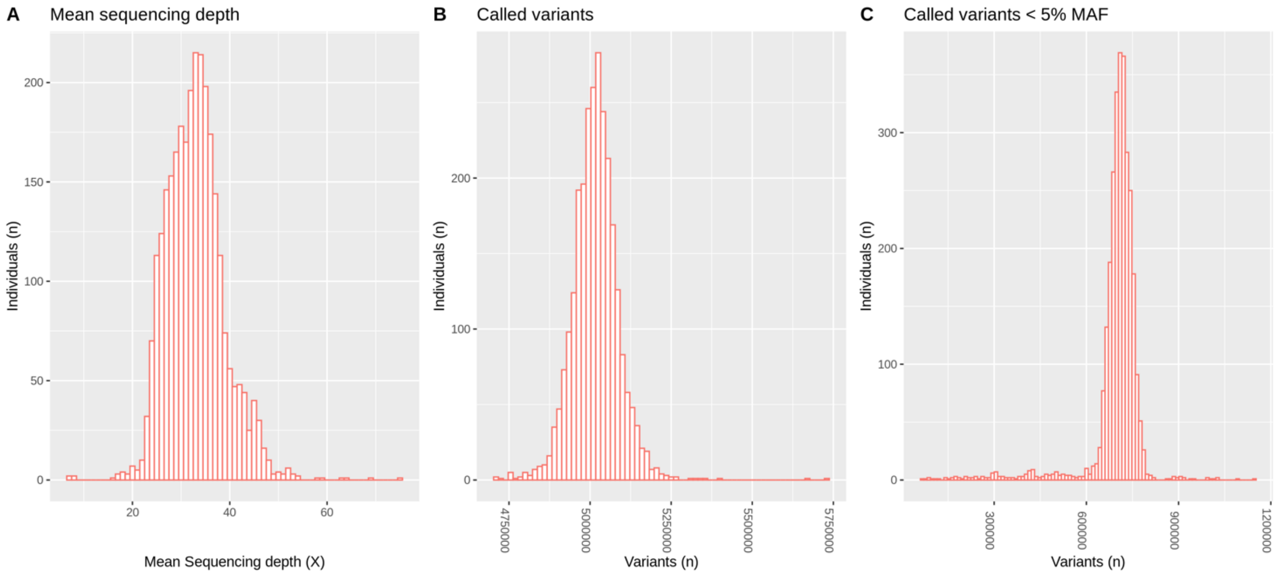

Supplementary figure 1. A) Histogram of mean sequencing depth of called variants per individual. The mean sequencing depth for all individuals is 32.94X ± 0.22X. **B-C)** Histogram of called variants per individual and of called variants < 5% MAF. Mean of called variants is 5017453 ± 2607 and mean of called variants < 5% MAF is 697044 ± 3244.

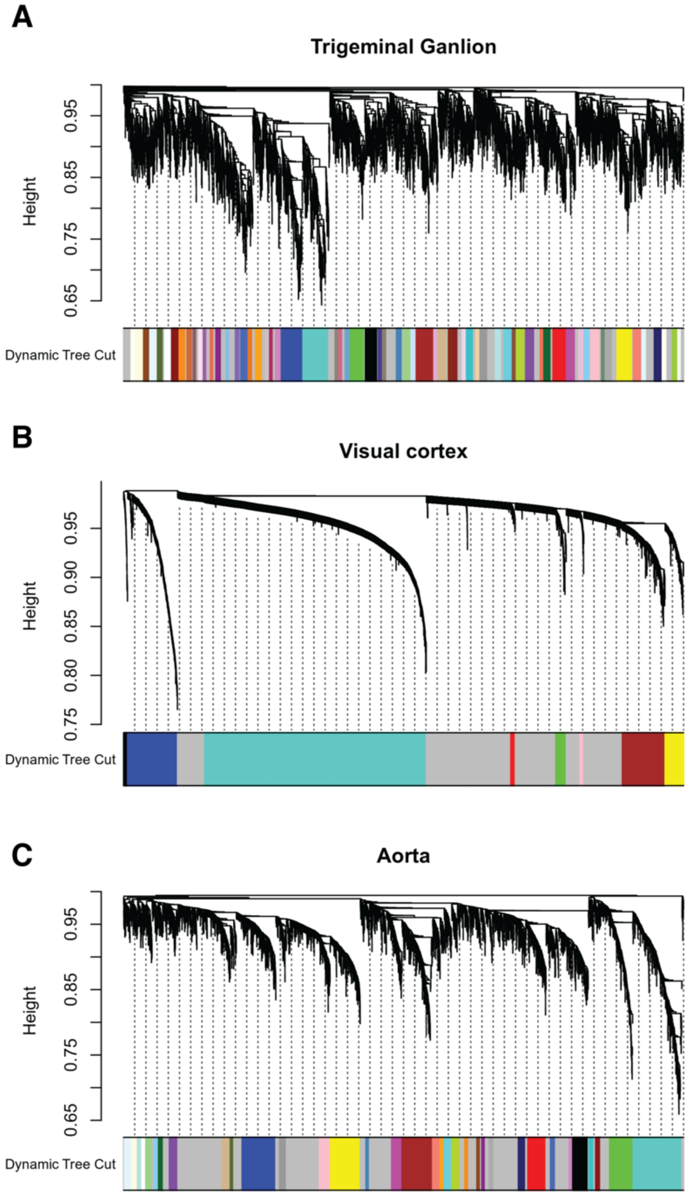

Supplementary figure 2. A-C) Gene dendrograms, with gene dissimilarity based on topological overlap on the y-axis, of the TG network, VC network and Aorta network. Clustering using the dynamic tree cutting algorithm results in 64, 8 and 34 modules, respectively, represented by colors on the x-axis.

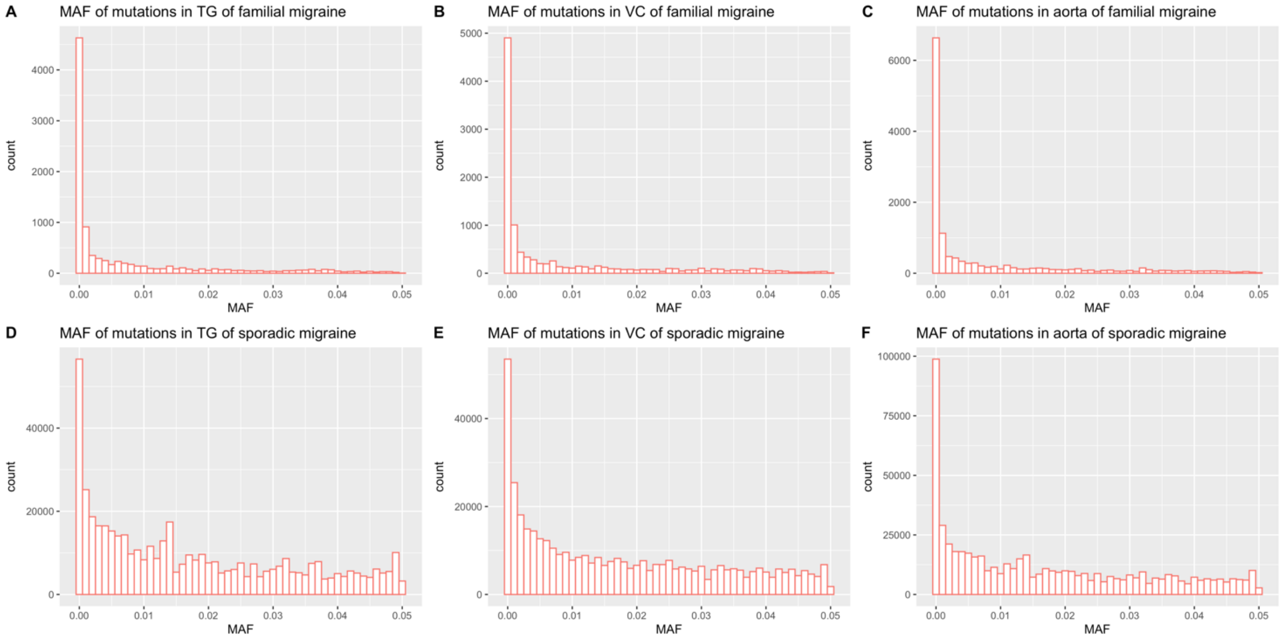

Supplementary figure 3. A-C) MAF distribution of mutations called in familial migraine for TG, VC and aorta networks. This distribution is from variants in all network genes before exclusion based on RVIS data. **D-F)** MAF distribution of mutations called in sporadic migraine for TG, VC and aorta networks. This distribution is from variants in all network genes before exclusion based on RVIS data.

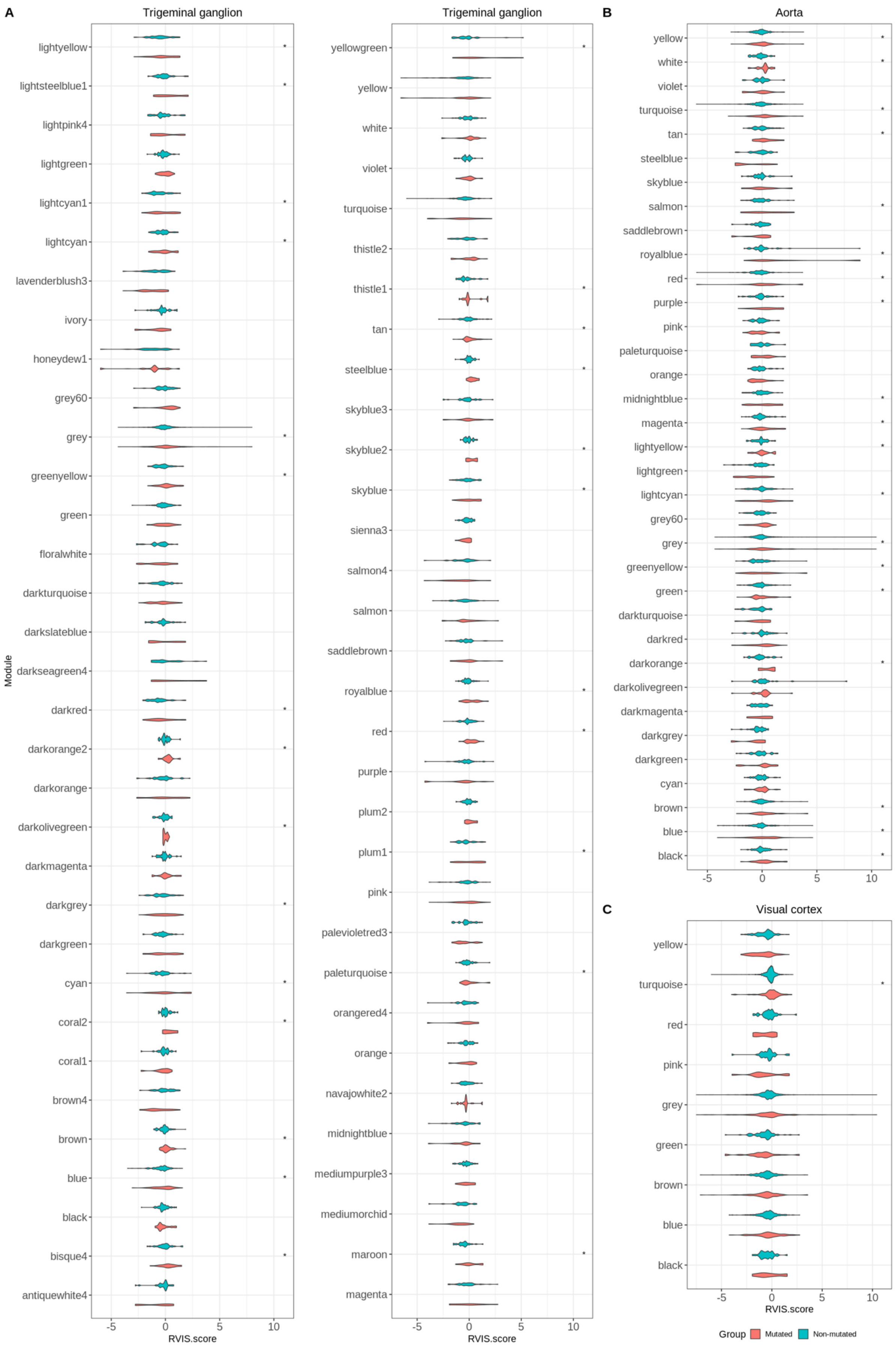

A
Trigeminal ganglion
lightyellow
lightsteelblue1
lightpink4
lightgreen
lightcyan1
lightcyan
lavenderblush3
ivory
honeydew1
grey60
grey
greenyellow
green
floralwhite
darkturquoise
darkslateblue
darkseagreen4
darkred
darkorange2
darkorange
darkolivegreen
darkmagenta
darkgrey
darkgreen
cyan
coral2
coral1
brown4
brown
blue
black
bisque4
antiquewhite4
Module
-5
0
5
10
RVIS.score
Trigeminal ganglion
yellowgreen
yellow
white
violet
turquoise
thistle2
thistle1
tan
steelblue
skyblue3
skyblue2
skyblue
sienna3
salmon4
salmon
saddlebrown
royalblue
red
purple
plum2
plum1
pink
palevioletred3
paleturquoise
orangered4
orange
navajowhite2
midnightblue
mediumpurple3
mediumorchid
maroon
magenta
-5
0
5
10
RVIS.score
B
Aorta
yellow
white
violet
turquoise
tan
steelblue
skyblue
salmon
saddlebrown
royalblue
red
purple
pink
paleturquoise
orange
midnightblue
magenta
lightyellow
lightgreen
lightcyan
grey60
grey
greenyellow
green
darkturquoise
darkred
darkorange
darkolivegreen
darkmagenta
darkgrey
darkgreen
cyan
brown
blue
black
-5
0
5
10
C
Visual cortex
yellow
turquoise
red
pink
grey
green
brown
blue
black
-5
0
5
10
RVIS.score
Group
Mutated
Non-mutated

Supplementary figure 4. A-C) Violinplot of mean RVIS score for mutated and non-mutated genes per module for TG, VC and Aorta. Asterisks display significant difference $P < 0.05$ between mutated and non-mutated genes.

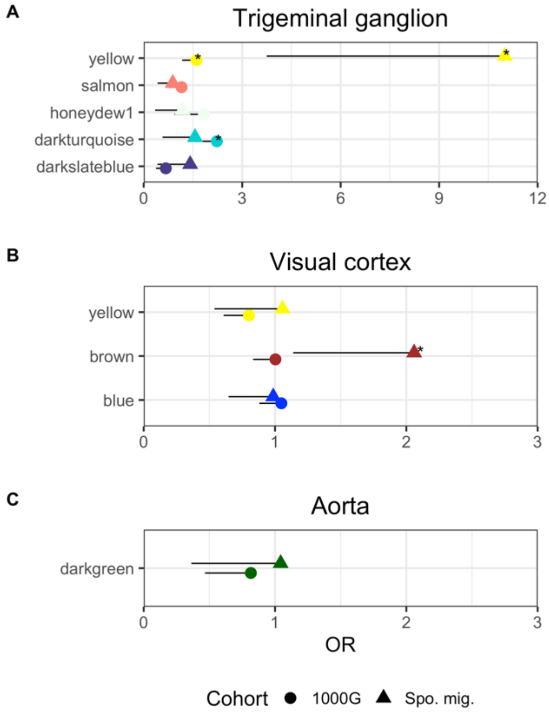

Supplementary figure 5. A-C) Replication of migraine-associated modules in sporadic migraine compared to 1000G with matched ethnicity, non-Finish Europeans. The odds ratio (OR) is displayed for each module and the error bars visualize the 95% confidence interval.

Supplementary table 1. The excel table contains three sheets with TG, VC and aorta network genes as well as three sheets with the mutated TG, VC and aorta genes in families after exclusion based on RVIS data. Finally, the excel table contains a sheet with Fisher's test statistics and a table with the frequency of mutated genes across families for the VC brown module.

Supplementary table 2. Table of total number of mutations per tissue for familial migraine.

Tissue	N mutations in familial migraine before RVIS exclusion	N mutations in familial migraine after RVIS exclusion
Visual cortex	5077	3244
Trigeminal ganglion	4747	2289
Aorta	6257	946
